# Chios Mastic Gum Extract Enhances Antioxidant Defense in Zebrafish

**DOI:** 10.3390/ijms262311338

**Published:** 2025-11-24

**Authors:** Pelagia Anastasiadou, Martina Samiotaki, Theoni Margaritopoulou, Kyriaki Machera, Konstantinos M. Kasiotis

**Affiliations:** 1 Laboratory of Pesticides’ Toxicology, Scientific Directorate of Pesticides Control and Phytopharmacy, Benaki Phytopathological Institute, 8 Stefanou Delta Str., 14561 Kifissia, Greece; b.anastasiadou@bpi.gr (P.A.); k.machera@bpi.gr (K.M.); 2 Βiomedical Sciences Research Center “Alexander Fleming”, 16672 Vari, Greece; 3 Laboratory of Mycology, Scientific Directorate of Phytopathology, Benaki Phytopathological Institute, 14561 Kifissia, Greece; th.margaritopoulou@bpi.gr

**Keywords:** Chios mastic gum, zebrafish, proteomics, copper-zinc superoxide dismutase, thioredoxin-disulfide reductase, catalase

## Abstract

Chios mastic gum (CMG), derived from the resin of the *Pistacia lentiscus* has long been considered a natural remedy in the Mediterranean region. Its anti-inflammatory and antioxidant properties have garnered increasing attention from scientists and consumers over recent decades. While substantial evidence supports CMG’s efficacy in preventing and treating common health disorders and its potential as a cancer cell inhibitor, the underlying molecular mechanisms remain poorly understood. In this study, we utilized zebrafish embryos as a model organism to identify molecular pathways modulated by CMG treatment. Embryos were exposed to non-toxic CMG concentrations for 3 to 96 h post-fertilization. LC-HRMS proteomics, combined with enrichment analysis, revealed oxidative phosphorylation (OxPhos), electron transport chain (ETC), and tricarboxylic acid cycle (TCA) as main processes. The latter highlights the benefits of CMG administration in energy generation and cytoskeletal integrity. From the plethora of identified proteins, hierarchical clustering revealed three main antioxidant proteins as upregulated, namely copper-zinc superoxide dismutase, thioredoxin-disulfide reductase, and catalase, confirming the contribution of CMG to the enhancement of zebrafish’s antioxidant defense.

## 1. Introduction

CMG is a natural resin obtained from the plant *Pistacia lentiscus* var. *chia*, which grows exclusively in southern Chios Island, in Greece [1]. CMG has been traditionally used in Greece since ancient times as an herbal medicine for various gastrointestinal disorders. In modern times, CMG is primarily used in the Mediterranean basin, but its applications have expanded as a dietary/nutritional additive in many everyday products [2]. In addition to its efficient use against minor digestive disorders, CMG also provides significant benefits for oral health [3].

Today, CMG’s antimicrobial and antifungal properties are being intensively exploited by the pharmaceutical and medicinal industries. Since 2014, CMG has been included in the European Medicines Agency list for treating skin disorders, minor wounds, and gastrointestinal conditions [4]. CMG is also active against *Helicobacter pylori*, helping cure peptic ulcers even at low doses [5,6,7]. Furthermore, there is evidence supporting CMG’s ability to prevent the oxidation of LDL, reducing the risk of atherosclerosis [8]. Other studies have highlighted its anti-inflammatory and antioxidant properties [9]. In the same context, the anticancer activity of CMG resin extracts has been reported in various tumor types [10,11,12], whereas Blomquist and Fernandez reviewed CMG’s potential benefits for cardiometabolic health [13].

Although the evidence supporting the health benefits of this natural product continues to grow, further research is needed, particularly regarding how administered doses affect protein expression. Most research has been focused on in vitro systems, so experiments conducted at the organism level are crucial for assessing CMG’s mode of action. One excellent model organism for performing developmental and toxicological studies in a whole-animal system, with direct comparisons to other vertebrates, including humans, is the zebrafish (*Danio rerio*). Zebrafish have been widely used as a model system in developmental biology, molecular genetics, and host–microbe–chemical interactions [14]. Additionally, zebrafish embryos are an invaluable model for examining the toxicity of substances (both chemical and natural) and assessing novel drugs in vivo [15].

In this context, the scrutiny on biological pathways benefited from CMG administration in zebrafish, and its effects on key antioxidant proteins (since CMG has already been linked to protection of LDL particle [8]), such as Copper-Zinc SuperOxide Dismutase (Cu-Zn-SOD or SOD1) and Catalase (Cat), are of particular interest. SOD1 plays a vital role in neutralizing superoxide radicals (O_2_^−^) by converting them into hydrogen peroxide (H_2_O_2_) and oxygen (O_2_), thus mitigating oxidative damage. Catalase further detoxifies H_2_O_2_ by breaking it down into water and oxygen, preventing oxidative stress. Studying the activity and expression of these enzymes in zebrafish following CMG exposure could provide essential insights into the potential antioxidant properties of CMG, leveraging its potential therapeutic applications.

## 2. Results and Discussion

**Figure 1:**
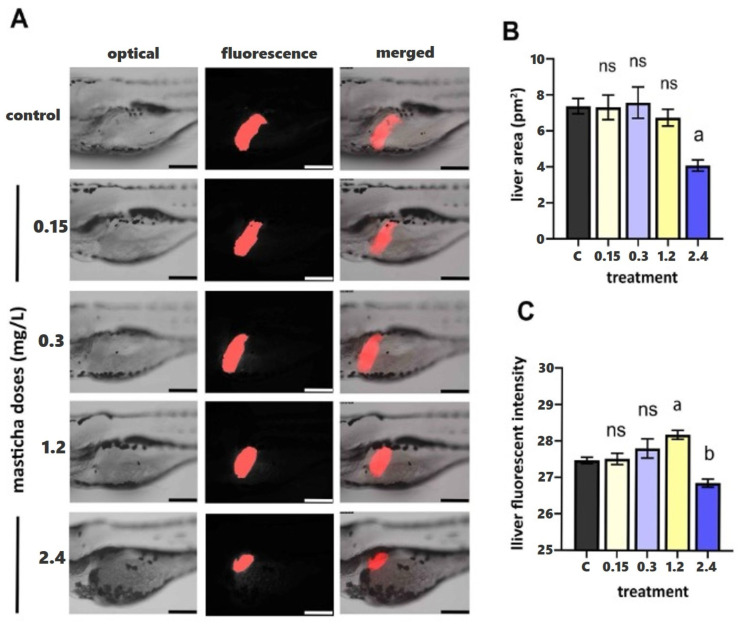
(**A**) Fluorescent stereoscopy of transgenic Tg(fabp10a: DsRed) zebrafish embryos after exposure to CMG at 0.15, 0.3, 1.2 and 2.4 mg/L, and 0.1% DMSO (control, 0 mg/L CMG) at 96 hpf. FABP distribution in the liver is reported in red. (**B**,**C**) Liver area (**B**) and liver fluorescent intensity (**C**) were calculated using Las X software (Leica Application Suite X). Data are expressed as mean ± SEM (*n* = 10) for all tested doses. Statistical analysis was performed with one-way ANOVA and Tukey’s post hoc multiple comparison test, *p* < 0.05. a,b, indicate statistical significance, ns: non-significant.

Mass spectrometry-based quantitative proteomics was performed to understand the changes induced by the administration of the CMG extract to the zebrafish embryos. We used the whole protein extracts of the embryos and sought to focus on the liver-related protein changes, as already observed microscopically (see Figure 1 below). Liver was selected due to its key roles in glycogen and lipid storage (interrelated with antioxidant activity), the metabolism of xenobiotics, and the zebrafish liver’s ability to regenerate and respond to stressors, as evidenced by liver histopathology [16,17]. The zebrafish cultures were fed with non-toxic CMG concentrations ranging from 0.15 to 2.4 mg/L for 96 h. The zebrafishes were pooled, homogenized, and processed with the protease trypsin.

### 2.1. Microscopic Investigation

Microscopic investigation (both optical and fluorescence, shown in Panel A in Figure 1, representative images from each treatment group, showing optical, fluorescence, and merged views) did not show significant differences in liver area between the treated groups and the control group at concentrations up to 1.2 mg/L.

More specifically, in the control and low-dose groups (0.15 and 0.3 mg/L), the liver displays a normal size and intense, homogeneous fluorescence, indicating unaltered morphology and hepatocyte viability. At 1.2 mg/L, a slight reduction in fluorescence intensity is observed, while liver boundaries remain well defined. Exposure to the highest concentration (2.4 mg/L) results in a visibly smaller and less fluorescent liver region, suggesting slight impairment of hepatocyte metabolism and reduced overall liver function. The quantification of liver area (pm^2^, Panel B, Figure 1) showed that doses up to 1.2 mg/L did not significantly affect liver size compared with the control. However, at 2.4 mg/L, a statistically significant reduction (*p* < 0.05) was detected. Panel C (Figure 1) presents the corresponding liver fluorescence intensity values, reflecting metabolic activity and hepatocyte integrity. Fluorescence remained stable at lower doses but significantly declined to 2.4 mg/L, corroborating the morphological findings.

Overall, most non-toxic CMG doses did not cause morphological changes in liver structure. To further explore the potential benefits of CMG administration, mass spectrometry-based proteomics was implemented.

### 2.2. Mass Spectrometry-Based Proteomics

#### 2.2.1. Protein Analysis

**Figure 2:**
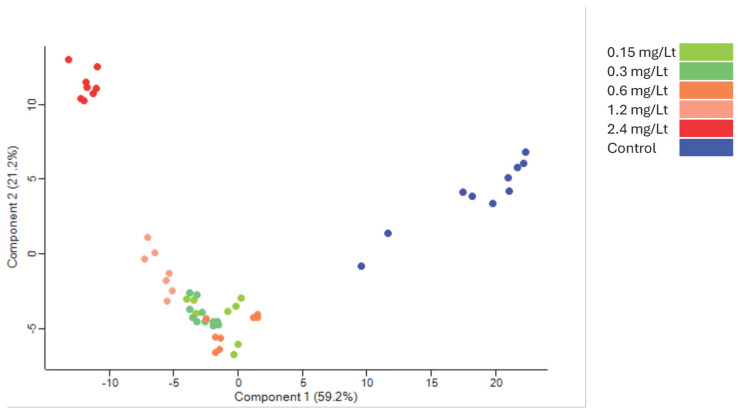
Principal component analysis of the grouping after the ANOVA test. Control in blue clearly separates from the groups receiving various CMG extract dosages (red corresponds to 2.4 mg/L, while the other colors represent intermediate levels).

The tryptic peptides were analyzed with nanoLC-MS/MS operating in DIA mode, generating a robust and sensitive dataset. The raw files were processed and the proteome identified was cross-run normalized within the DIA-NN software. The generated dataset consisted of a total of almost 10,500 protein groups and 8300 proteins identified using only proteotypic peptides. This high-quality dataset nearly encompasses the total canonical proteome of zebrafish embryos (see Section 3, Supplementary Data S1). Principal component analysis (PCA) presented a distinct separation between extract dosing (Figure 2).

**Figure 3:**
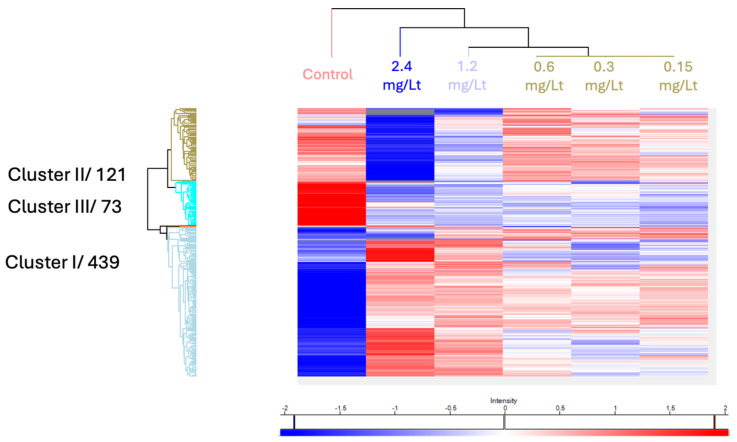
The 444 proteins that were found significantly deregulated after an ANOVA test were visualized using hierarchical clustering.

Global comparison between the various doses visualized by a heat map, displayed major dose-dependent changes that were clustered into three main groups (see Figure 3). The effect on the proteome level was more prominent at the two highest concentrations.

To investigate the associated biological pathways that were potentially overexpressed in the large proteomic dataset, enrichment analysis was applied.

#### 2.2.2. Enrichment Analysis

**Figure 4:**
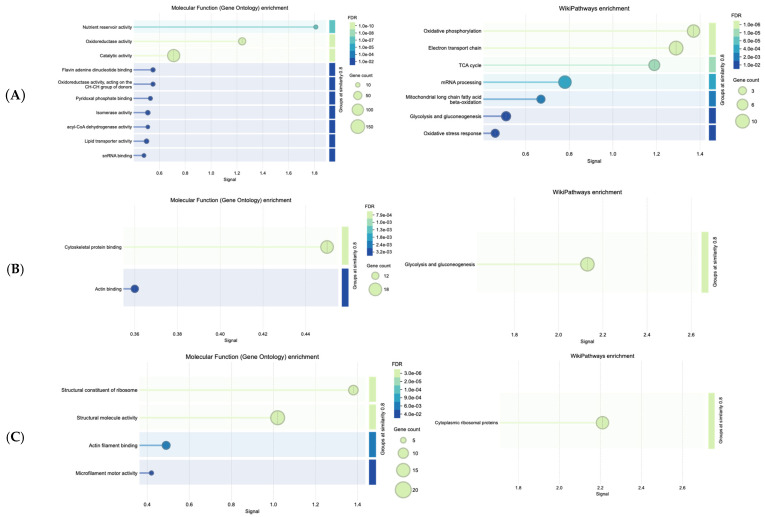
Wikipathways related to increased dosage effect and molecular functions of proteins in distinct clusters: (**A**), Cluster I, (**B**), Cluster II, (**C**), Cluster III.

The STRING databases mediated enrichment analysis (false discovery rate (FDR) values are presented in the Wikipathways diagrams, Figure 4A, Figure 4B (FDR = 5.35 × 10^−9^) and Figure 4C (FDR = 5.59 × 10^−9^), respectively, and signal in x-axis represents the enrichment score) of the three main clusters, revealing Wikipathways primarily related to OxPhos, ETC, and TCA, and to a lesser extent to mitochondrial long-chain fatty acid beta-oxidation (MLCFA beta-oxidation) and mRNA processing (to mature RNA).

Natural products and their constituents are described as modulators of mitochondrial functions [18]; yet, mitochondria (indicatively, Q9PUL9 protein was identified, known as mitochondrial carrier homolog) are essential for OxPhos (known to be necessary for cellular respiration) among others. The overexpression of the MLCFA beta-oxidation pathway favors the breakdown of long-chain fatty acids and eventually metabolic energy generation. Defects of MLCFA beta-oxidation are linked to health disorders, such as cardiomyopathy and arrhythmias [19]. From the obtained results, it was observed that CMG extract’s dose-dependent increase interrelates with the enzymatic pathways of OxPhos, TCA cycle, and ETC. For Cluster I, the molecular function of proteins (Figure 4A) was epitomized by the nutrient reservoir activity, which suggests that CMG administration may be involved in the storage and release of nutrients and oxidoreductase activity, pivotal for electron transfer. Similarly, from an energy perspective, Cluster II revealed the role of CMG in regulating the glycolysis and gluconeogenesis pathways (Figure 4B), which is in line with other natural products reported to be pivotal in regulating glycolysis, a process also essential for a therapeutic purposes, even in malignancies [20]. Cytoskeletal architecture (cytoskeletal protein binding, CPB), which emerged from the molecular functions (Figure 4B), is known to be mechanistically involved in the regulation of glycolysis [21]. Actin binding enhancement, even though not as pronounced as CPB, corroborates cytoskeleton integrity [22], which is essential for all organisms.

Ribosome biogenesis and protein synthesis (see Figure 4C) are crucial limiting steps for cell growth and proliferation. The molecular functions of Cluster III (Figure 4C) suggest that CMG administration contributes to the structural integrity of ribosomes (including their subunits), which can be viewed as an advantage of this natural product, given that ribosome protection helps prevent ribosomopathies linked to phenotypic abnormalities and cancer [23]. Explorations of natural products have shown clear benefits on key processes of RNA translation, involving ribosomes [24].

#### 2.2.3. Antioxidant Proteins Upregulation

**Figure 5:**
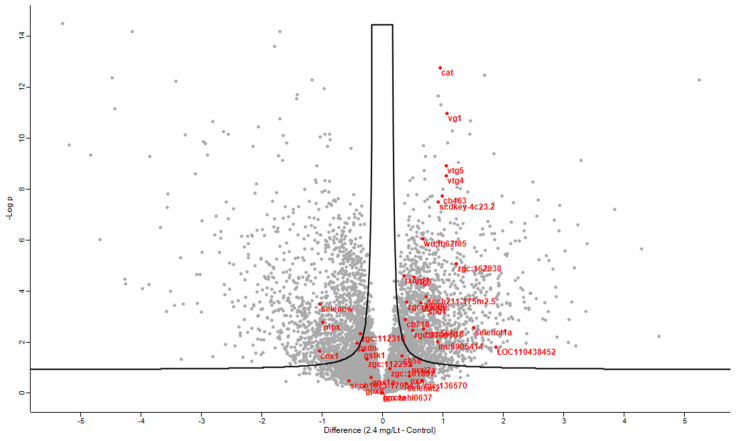
Volcano plot of significantly regulated proteins at 5% FDR when comparing control proteomes to proteomes after incubation with the highest dosing of Mastiha extract. Red represents proteins with antioxidant activity.

**Figure 6:**
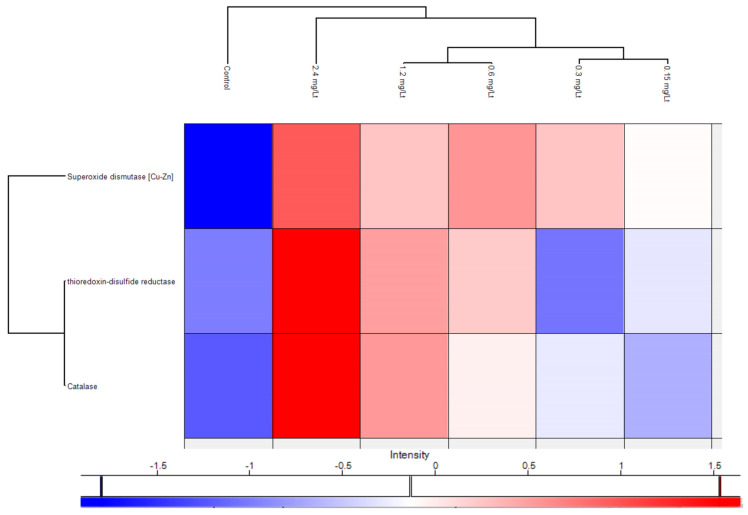
Heat map of three main antioxidant proteins showing their gradual increase in dependence of the CMG extract dosing.

In the same context, and to provide a refined and in-depth view of key regulated proteins in parallel, a plethora of antioxidant proteins were significantly regulated after CMG administration, as portrayed in the volcano plot below (Figure 5). To draw a firmer conclusion about antioxidant proteins in CMG, a heat map was generated showing the relationship between the three primary antioxidant proteins’ levels and the dose-dependent increase (Figure 6).

Notably, the upregulation of the metalloenzyme of SOD1 known for its antioxidant defense profile (due to inactivation of the superoxide radical) and therapeutic potential [25,26] indicates the beneficial effect of CMG administration. In the same context, SOD1 is also reported as a potential inhibitor of inflammation [27]. Mechanistic details of increased SOD activity in relation to natural products entail direct binding with hydrogen bonds and van der Waals forces, as demonstrated by Ma and coworkers in an in vitro study [28], while conformational shifts were reflected in increased α-helix content, and modulation of redox-sensitive and immune pathways. Tissue-specific effects were documented in liver [29], lung, cerebral cortex, and other sites. These reports consistently indicate that natural products from plant, bacterial, and mineral sources upregulate or restore Cu/Zn-SOD activity through diverse molecular mechanisms. The increase in thioredoxin-disulfide reductase, known for its capacity to regenerate reduced thioredoxin (thioredoxins are pivotal for the redox regulation of proteins) [30], with the elevation of CMG doses, adds further evidence for the antioxidant capacity of CMG. Another upregulated main antioxidant protein was Cat, present in the majority of aerobic organisms. Cat is known for its ability to prevent the accumulation of peroxide and to break it down [31], underpinning the antioxidant potential of CMG. From a chemical perspective, triterpenoids, exemplified by isomasticadienonic acid, masticadienonic acid, moronic and oleaonic acid, are known constituents of CMG, accounting for almost 70% of its composition, recently corroborated by our team as well using an LC-HRMS platform [32,33,34]. Triterpenes are reported as scavengers of reactive oxygen species (ROS) [35,36]. Similarly, the phenolic compounds present in CMG (at lower abundance compared to triterpenoids) are likely related to the enhancement of its antioxidant potential [37]. Apart from the aforementioned major antioxidant proteins, additional proteins emerged after an in-depth exploration of the proteome. Specifically, the overexpressed apoliprotein A-I (principal constituent of high-density lipoproteins) has a fundamental role in inhibiting oxidative damage by reducing lipid peroxide levels and scavenging harmful compounds [38]. Pyruvate kinase upregulation documented in this work is linked to cellular antioxidant defense, as notably demonstrated for its M2 isoform [39]. Phosphopyruvate hydratase, though also not directly associated with antioxidant activity, is a glycolysis-regulating enzyme involved in oxidative stress responses; therefore, its increase can be valuable for organisms [40]. Similarly, serotransferrin upregulation indicates the beneficial effects of CMG on iron homeostasis and, through iron binding, the lowering of free iron levels, which are essential for the replication of microbia and bacteria. In the same direction, Fraenkel and coworkers showed that transferrin-α played a key role in iron transport across zebrafish embryos and in hepcidin regulation [41]. Last but not least, the work presented herein also disclosed the upregulation of annexins (see Supplementary Material), proteins known for their involvement in anti-inflammatory processes and the protection of cells from phagocytosis [42]. Gioxari and coworkers reported that Chios mastic essential oil, rich in monoterpenes, demonstrates anti-obesity effects potentially linked to the modulation of inflammatory and antioxidant processes [43]. Scaling and cross-species extrapolation of the observed findings needs caution. Specifically, in this work, attempts to investigate and infer potential effects on humans have a high probability of risk, since the administered CMG extract (acidic fraction) is not directly comparable (from a chemical composition perspective) to the raw consumed CMG [44]. In the same context, such attempts require species-specific toxicokinetic and toxicodynamic modeling [45] which were out of the scope of the specific study. Overall, the proteomics findings of this work revealed the upregulation of specific antioxidant proteins, suggesting that the predominant constituents of CMG have a potentially beneficial impact on its antioxidant activity, ultimately fostering zebrafish’s antioxidant defense.

## 3. Materials and Methods

### 3.1. CMG Extract

CMG was provided by the Chios Mastiha Growers Association (Chios Island, Greece), the exclusive worldwide producer of the resin. The dried extract of the acidic fraction of mastic, containing the characteristic triterpenic acids of CMG, was utilized in this study. For the experiments, the extract was dissolved in 0.1% DMSO.

### 3.2. Zebrafish Breeding and Embryo Care

Zebrafish embryos were maintained under standard laboratory conditions at 28 °C. The genetic backgrounds used included the wild-type AB strain for all screenings and the Tg(fabp10a) transgenic line for liver development visualization (all zebrafish were acquired from the Human Disease Laboratory, Biomedical Research Foundation, Academy of Athens, Greece). All zebrafish maintenance procedures complied with the European Directive 2010/63 for the protection of animals used for scientific purposes and the Recommended Guidelines for Zebrafish Husbandry Conditions. Since the experimental protocols involved zebrafish larvae up to 96 h post-fertilization (hpf), they were exempt from European animal protection guidelines.

### 3.3. Zebrafish Embryonic Toxicity Test

The acute toxicity of CMG on zebrafish embryos was assessed according to the “Fish Embryo Acute Toxicity (FET) Test,” outlined in the OECD Guidelines for the Testing of Chemicals—Test No. 236 [46]. Newly fertilized zebrafish eggs were exposed to increasing concentrations of CMG for 96 hpf (hours post-fertilization). Each test included at least three concentrations of CMG, a solvent control (0.1% DMSO in egg water), and a negative control (egg water). Embryos were observed under a stereoscope at 24, 48, 72, and 96 hpf, and the following lethality indicators were recorded: (i) coagulation of fertilized eggs, (ii) absence of somite formation, (iii) failure of tail-bud detachment from the yolk sac, and (iv) absence of a heartbeat. At the end of the exposure period, the 50% lethal concentration (LC50) was calculated. Morphological abnormalities were also documented.

#### Range-Finding Test

As no prior data on CMG toxicity to zebrafish were available, a range-finding test was initially performed to determine the concentration range, spanning from 1 to 150 mg/L. For each dose in the range-finding test, at least 20 embryos were used. The experiment was repeated twice with three independent replicates. LC50 values were determined using Probit analysis via SPSS software (version 16).

### 3.4. Fluorescent Stereoscopy of Liver Development

Fertilized eggs of the zebrafish transgenic Tg(fabp10a) line were incubated for 96 h post-fertilization (hpf) with various concentrations of CMG. After exposure, embryos were fixed in paraformaldehyde solution (4% in PBS) overnight at 4 °C. The fixed embryos were then mounted on slides for observation. The Tg(fabp10a) line features liver-specific DsRed expression driven by the fabp10a promoter, allowing visualization of the liver using fluorescent stereoscopy. Liver fluorescence intensity was assessed using a Leica M165 FC stereoscope (Wetzlar, Germany), and images were analyzed with LAS X (Leica) software to quantify fluorescence in the liver area.

### 3.5. Bioassay for Liver Effects Assessment in Zebrafish Embryos

Fertilized eggs from transgenic zebrafish [Tg(lfabp10)] were incubated immediately after fertilization for 96 h in either the tested extracts or egg water (control group). After incubation, embryos were fixed with paraformaldehyde (4% in PBS, overnight at 4 °C), placed on slides, and observed using a fluorescence stereoscope. Liver cells were labeled with the red fluorescent dye (mCherry, Biosynth, UK), allowing for the calculation of liver area and fluorescence intensity using appropriate software. These parameters were compared between the control and treated groups, with statistically significant differences in liver size or fluorescence intensity serving as indicators of liver toxicity caused by exposure to the tested extract.

### 3.6. LC50 Determination

The median lethal concentration (LC50) of CMG on zebrafish embryos was determined through Probit analysis, using SPSS software (version 16). The LC50 was calculated based on mortality rates observed over a 96 h exposure period to varying concentrations of CMG.

The estimated LC50 value for CMG was 6.001 mg/L (95% confidence interval: 5.453 to 6.583 mg/L), as shown in Table S1. The Pearson goodness-of-fit test for the Probit model yielded a chi-square value of 0.035 with 2 degrees of freedom and a significance level of 0.982, indicating that the model adequately fits the data (Table S2). No heterogeneity factor was applied to the calculation of confidence limits, as the significance level was greater than 0.150.

### 3.7. Proteomic Analysis

#### 3.7.1. Proteomic Sample Preparation Using the Single-Pot, Solid-Phase-Enhanced Sample Preparation Sp3-Mediated Protein Digestion Protocol

The zebrafish samples were homogenized and the cells were lysed in a buffer containing 4% SDS and 0.1 M DTT. The homogenate was heated, sonicated and centrifuged. The supernatant was collected and processed according to the Sp3 protocol ^(Hughes)^ including an alkylation step in the dark for 15 min in 10 mg/mL iodoacetamide (Acros Organics, Geel, Belgium). A quantity of 20 μg of beads (1:1 mixture of hydrophilic and hydrophobic SeraMag carboxylate-modified beads, GE Life Sciences, Marlborough, MA, USA) was added to each sample in 50% ethanol. The proteins were allowed to bind to the beads for 15 min followed by repeated steps of protein clean-up using a magnetic rack. The beads were washed two times with 80% ethanol and once with 100% acetonitrile (Fisher Chemical, Waltham, MA, USA). The captured beads proteins were digested overnight at 37 °C under vigorous shaking (1200 rpm, Eppendorf Thermomixer, Hamburg, Germany) with 0.5 ug Trypsin/LysC (MS grade, Promega, Madison, WI, USA) prepared in 25 mM ammonium bicarbonate.

##### Peptides Purification and Concentration Determination

The next day, the supernatants were collected and the peptides were purified using a modified Sp3 clean-up protocol and finally solubilized in the mobile phase A (0.1% formic acid in water) and sonicated, and the peptide concentration was determined through absorbance at 280 nm measurement using a nanodrop instrument.

#### 3.7.2. LC-MS/MS Analysis

Samples were run on a liquid chromatography–tandem mass spectrometry (LC-MS/MS) setup consisting of a Dionex UltimateRSLC online with a Thermo Q Exactive HF-X Orbitrap mass spectrometer (Thermo Scientific, Waltham, MA, USA). Peptidic samples were directly injected and separated on a 25 cm-long analytical C18 column (PepSep, 1.9 μm^3^ beads, 75 µm ID, Bruker, Billerica, MA, USA) using a one-hour long run, starting with a gradient consisting of 7% Buffer B (0.1% Formic acid in 80% Acetonitrile) to 35% for 40 min and followed by an increase to 45% in 5 min and a second increase to 99% in 0.5 min and then kept constant for equilibration for 14.5 min. The flow rate was set to 400 nL/min in the sample loading phase and lowered to 250 nL/min in the main phase of sample analysis, A full MS was acquired in profile and positive mode using a mass spectrometer, operating in the scan range of 375–1400 *m*/*z* using 120K resolving power with an AGC of 3 × 10^6^ and max IT of 60 ms followed by data independent analysis using 8 Th windows (39 loop counts) with 15K resolving power with an AGC of 3 × 10^5^ and max IT of 22 ms and a normalized collision energy (NCE) of 26. At least two technical replicas were acquired per sample.

#### 3.7.3. Data Analysis

Orbitrap raw data were analyzed in DIA-NN 1.8.1 (Data-Independent Acquisition by Neural Networks) ^Demichev^ through searching against the canonical *Danio rerio* (Zebrafish) database downloaded from UniProt using the library-free mode of the software and allowing up to two tryptic missed cleavages (detailed DIA-NN parameters are provided in the Supplementary Material). A spectral library was created from the DIA runs and used to reanalyze them. DIA-NN default settings have been used with oxidation of methionine residues and acetylation of the protein N-termini set as variable modifications and carbamidomethylation of cysteine residues as fixed modification. N-terminal methionine excision was also enabled. The match between runs (MBR) feature was used for all analyses and the output (precursor) was filtered at 0.01 FDR. Finally, the protein inference was performed on the level of genes using only proteotypic peptides. The generated results were processed statistically and visualized in the Perseus software (1.6.15.0) ^Tyanova^. Intensity values were log(2) transformed, a threshold of 70% of valid values in at least one group was applied and the missing values were replaced from a normal distribution. For statistical analysis, both ANOVA and Student’s *t*-test were performed and permutation-based FDR values were calculated.

Enrichment analysis of deregulated proteins was performed on the Genecodis website.

#### 3.7.4. Data Deposition

The mass spectrometry proteomics data have been deposited to the ProteomeXchange Consortium via the PRIDE partner repository [47], with dataset identifier PXD070358.

## 4. Conclusions

In the presented work, a CMG extract was administered to zebrafish embryos to investigate the elicited changes at the proteome level. The latter was accomplished by applying mass spectrometry-based proteomics and gene ontology enrichment. The primary biological pathways that were affected were oxidative phosphorylation (OxPhos), electron transport chain (ETC), and tricarboxylic acid cycle (TCA), favoring both the cytoskeleton and energy generation. Among the multitude of upregulated antioxidant proteins, three main proteins were unveiled: copper-zinc superoxide dismutase, thioredoxin-disulfide reductase, and catalase, verifying the contribution of CMG to the enhancement of zebrafish’s antioxidant defense.

## Data Availability

The original contributions presented in this study are included in the article/Supplementary Material. Further inquiries can be directed to the corresponding authors.

## Supplementary Materials

The following supporting information can be downloaded at: https://www.mdpi.com/article/10.3390/ijms262311338/s1.
