# Supplementary material for: Chios Mastic Gum Extract Enhances Antioxidant Defense in Zebrafish
Source: Int J Mol Sci. 2025 Nov 24;26(23):11338. doi: 10.3390/ijms262311338 (PMC12692334; doi:10.3390/ijms262311338)
Supplement: Supplementary file 1 [file ijms-26-11338-s001.zip › Supplementary Material Tables.pdf]

Supplementary Material

Chios Mastic Gum Extract Enhances Antioxidant Defense in Zebrafish

Pelagia Anastasiadou, Martina Samiotaki, Theoni Margaritopoulou, Kyriaki Machera and Konstantinos M. Kasiotis

Table S1. Confidence limits in LC50 determination

Confidence Limits

	Probability	95% Confidence Limits for dose			95% Confidence Limits for log(dose)[a]		
		Estimate	Lower Bound	Upper Bound	Estimate	Lower Bound	Upper Bound
PROBIT	,010	4,015	2,971	4,616	,604	,473	,664
	,020	4,209	3,208	4,785	,624	,506	,680
	,030	4,337	3,368	4,897	,637	,527	,690
	,040	4,435	3,492	4,984	,647	,543	,698
	,050	4,517	3,596	5,056	,655	,556	,704
	,060	4,588	3,687	5,119	,662	,567	,709
	,070	4,651	3,767	5,175	,668	,576	,714
	,080	4,708	3,841	5,227	,673	,584	,718
	,090	4,761	3,909	5,274	,678	,592	,722
	,100	4,810	3,972	5,319	,682	,599	,726
	,150	5,018	4,242	5,512	,700	,628	,741
	,200	5,189	4,465	5,677	,715	,650	,754
	,250	5,341	4,660	5,828	,728	,668	,766
	,300	5,482	4,837	5,974	,739	,685	,776
	,350	5,615	5,002	6,119	,749	,699	,787
	,400	5,744	5,158	6,267	,759	,713	,797
	,450	5,872	5,308	6,420	,769	,725	,808
	,500	6,001	5,453	6,583	,778	,737	,818
	,550	6,133	5,596	6,757	,788	,748	,830
	,600	6,270	5,738	6,947	,797	,759	,842
	,650	6,414	5,882	7,158	,807	,770	,855
	,700	6,570	6,029	7,396	,818	,780	,869
	,750	6,743	6,185	7,672	,829	,791	,885
	,800	6,940	6,355	8,001	,841	,803	,903
	,850	7,178	6,549	8,415	,856	,816	,925
	,900	7,488	6,791	8,982	,874	,832	,953
	,910	7,565	6,849	9,126	,879	,836	,960
	,920	7,650	6,912	9,287	,884	,840	,968
	,930	7,744	6,981	9,467	,889	,844	,976
	,940	7,850	7,059	9,674	,895	,849	,986
	,950	7,974	7,148	9,916	,902	,854	,996
	,960	8,121	7,253	10,210	,910	,860	1,009
	,970	8,305	7,382	10,586	,919	,868	1,025
	,980	8,557	7,556	11,110	,932	,878	1,046
	,990	8,970	7,833	11,995	,953	,894	1,079

a. Logarithm base = 10.

Table S2. Pearson goodness-of-fit test for the Probit model

Chi-Square Tests

		Chi-Square	df[a]	Sig.
PROBIT	Pearson Goodness-of-Fit Test	,035	2	,982[b]

a. Statistics based on individual cases differ from statistics based on aggregated cases.

b. Since the significance level is greater than ,150, no heterogeneity factor is used in the calculation of confidence limits.

DIA-NN 1.8.1 (Data-Independent Acquisition by Neural Networks)

Thread number set to 20

Output will be filtered at 0.01 FDR
Precursor/protein x samples expression level matrices will be saved along with the main report
A spectral library will be generated
Deep learning will be used to generate a new in silico spectral library from peptides provided
Library-free search enabled
Min fragment m/z set to 200
Max fragment m/z set to 1800
N-terminal methionine excision enabled
In silico digest will involve cuts at K*,R*
Maximum number of missed cleavages set to 2
Min peptide length set to 7
Max peptide length set to 30
Min precursor m/z set to 300
Max precursor m/z set to 1800
Min precursor charge set to 1
Max precursor charge set to 4
Cysteine carbamidomethylation enabled as a fixed modification
Maximum number of variable modifications set to 3
Modification UniMod:35 with mass delta 15.9949 at M will be considered as variable
Modification UniMod:1 with mass delta 42.0106 at *n will be considered as variable
Neural networks will be used for peak selection
Mass accuracy will be determined separately for different runs
Scan windows will be inferred separately for different runs
A spectral library will be created from the DIA runs and used to reanalyse them; .quant files will only be saved to disk during the first step
When generating a spectral library, in silico predicted spectra will be retained if deemed more reliable than experimental ones
Interference removal from fragment elution curves disabled
DIA-NN will optimise the mass accuracy separately for each run in the experiment. This is useful primarily for quick initial analyses, when it is not yet known which mass accuracy setting works best for a particular acquisition scheme.
Exclusion of fragments shared between heavy and light peptides from quantification is not supported in FASTA digest mode - disabled; to enable, generate an in silico predicted spectral library and analyse with this library
The following variable modifications will be scored: UniMod:1

Excel file (attached as a separate file)
